# Serial absolute lactate value < 4 versus relative 10% reduction as a predictor of mortality in severe sepsis and septic shock

**DOI:** 10.1186/2197-425X-3-S1-A359

**Published:** 2015-10-01

**Authors:** S Lokhandwala, P Patel, MN Cocchi, MW Donnino

**Affiliations:** Beth Israel Deaconess Medical Center, Internal Medicine, Boston, United States; Harvard Medical School, Boston, United States; Beth Israel Deaconess Medical Center, Emergency Medicine, Boston, United States; Beth Israel Deaconess Medical Center, Anesthesia Critical Care, Boston, United States; Beth Israel Deaconess Medical Center, Pulmonary and Critical Care, Boston, United States

## Introduction

Serial lactate measurements provide a valuable tool to risk-stratify patients, determine the presence of ongoing tissue hypoperfusion, and potentially evaluate efficacy of therapeutic interventions. Prior studies have shown lactate clearance to be a valuable predictor of patient outcomes, however some have defined adequate change in lactate by a decrease from baseline by 10% whereas others considered an absolute reduction to lactate < 4mmol/L. We hypothesize that a serial absolute lactate < 4mmol/L after initial resuscitation will be as effective a predictor of future mortality as a change by 10%.

## Objectives

To compare the diagnostic characteristics of an absolute serial lactate < 4 mmol/L after resuscitation with the more traditional definition (>10% decrease in serum lactate).

## Methods

Single-center retrospective study of patients presenting to an urban tertiary care Emergency Department (ED) with lactate >4mmol/L and suspected infection. Continuous data was analyzed using a one-way ANOVA, whereas categorical data was compared using Fisher's exact test. Patients were stratified by lactate clearance using the traditional definition (>10% decrease in serum lactate) as well as a novel definition (second lactate < 4mmol/L) and compared.

## Results

Median initial lactate was 5.2mmol/L [IQR 4.4-6.8]. The average decrease in serum lactate was 26.7% [2.3-44.4]. In-hospital mortality was 26.7%. 109/161 (67.7%) patients had >10% decrease in serum lactate, whereas 76/161 (47.2%) cleared lactate to < 4mmol/L. The average amount of crystalloid fluid resuscitation received was 2,970 ml, and the average time between lactate values was 181 minutes. Among patients who cleared lactate by at least 10%, mortality was 21.1% while those who did not clear their lactate by 10% had 38.5% mortality (p = 0.02). The negative predictive value for in-hospital mortality among those who clear their lactate by 10% was 78.9% [95% CI 69.8-85.9], sensitivity was 46.5% [95% CI: 31.5-62.2] and specificity was 72.9% [95% CI: 63.8-80.5]. Among patients who cleared their lactate to < 4mmol/L, mortality was 14.5%, while those who did not clear their lactate to < 4mmol/L had a mortality of 37.6% (p = 0.001). The negative predictive value for in-hospital mortality among those who clear lactate to < 4mmol/L was 85.5% [95% CI 75.2-92.2], sensitivity was 74.4% [58.5-86.0] and specificity was 55.1% [45.7-64.2].

## Conclusions

A serial lactate < 4mmol/L as compared to >10% change has improved negative predictive value for in-hospital mortality and may provide more utility for risk stratification, assessment of response to therapy, and potentially clinical decision-making.

## Grant Acknowledgment

Dr. Donnino, the Primary Investigator, is supported by NHLBI 1K02HL107447-01A1 and Dr. Cocchi is supported by AHA 15SDG22420010.

**Table 1:** Baseline Characteristics

	Lactate decrease <10% n = 52 (32.3)	Lactate decrease > 10% n = 109 (67.7)	Serial Lactate > 4mmol/L n = 85 (52.8)	Serial Lactate <4mmol/L n = 76 (47.2)	Overall
**Age**	67.2 + 18.4	69.3 +15.2	68.4+17.9	69.0+14.4	68.6+16.3
**Initial Lactate, mmol/L**	5.25 [4.4-7.1]	5.2 [4.4-6.7]	6 [4.9-8.6]	4.7 [4.3-5.3]	5.2[4.4-6.8]
**% Decrease in Lactate**	0.0 [-13.3-2.26]	37.3 [25.6-52.7]	3.9[-3.7-21.0]	44.4[32.4-58.1]	26.7[2.3-44.4]
**Time between lactate values, minutes**	168 +109.7	188+80.2	163+99.5	202+75.9	181+90.9
**Volume Crystalloid in 3 hours, ml**	2971+1931	2969+1499	3080+1865	2850+1358	2970+1640
**Initial Systolic Blood Pressure**	105+31.0	100+23.6	105.9+28.7	97.8+22.7	102.0+26.2
**Vasopressors Use within 3 hours**	22 (42.3)	32 (29.4)	35 (41.2)	19 (25.0)	54 (33.5)
**ICU LOS, days**	2.1 [1.1-4.6]	2.2 [1.3-5.9]	2.7 [1.2-5.9]	1.9[1.1-5.4]	2.2[1.1-5.7]
**In-Hospital Mortality**	20 (38.5)	23 (21.1)	32 (37.6)	11 (14.5)	43 (26.7)

**Table 2:** Comparison based on Lactate Clearance

	Lactate decrease <10%	Lactate decrease > 10%	P-value	Serial Lactate > 4mmol/L	Serial Lactate <4mmol/L	P-value
**N**	52	109	-	85	76	-
**Initial Lactate, mmol/L**	6.1	5.9	0.43	6.8	4.9	< 0.0001
**Volume Crystalloid in 3 hours, ml**	2971.7	2969.4	0.99	2850.5	3080.6	0.39
**Vasopressors within 3 hours**	22 (42.3)	32 (29.4)	0.11	35 (41.2)	19 (25.0)	0.04
**In-hospital Mortality**	20 (38.5)	23 (21.1)	0.02	32 (37.7)	11 (14.5)	0.001

